# First-degree family history of breast cancer is associated with prostate cancer risk: a systematic review and meta-analysis

**DOI:** 10.1186/s12885-019-6055-9

**Published:** 2019-09-02

**Authors:** Zheng-Ju Ren, De-Hong Cao, Qin Zhang, Peng-Wei Ren, Liang-Ren Liu, Qiang Wei, Wu-Ran Wei, Qiang Dong

**Affiliations:** 1 Department of Urology, Institute of Urology, West China Hospital, Sichuan University, 37, Guo Xue Road, Chengdu, 610041 China; 2 State Key Laboratory of Biotherapy and Cancer Center, Collaborative Innovation Center for Biotherapy, West China Hospital, Sichuan University, Chengdu, China; 3 Department of Radiology, Chongqing Traditional Chinese Medicine Hospital, Chongqing, China; 4 Department of Evidence-Based Medicine and Clinical Epidemiology, West China Hospital, Sichuan University, Chengdu, China

**Keywords:** Prostate cancer, Breast cancer, Family history, Meta-analysis

## Abstract

**Background:**

The relationship between first-degree family history of female breast cancer and prostate cancer risk in the general population remains unclear. We performed a meta-analysis to determine the association between first-degree family history of female breast cancer and prostate cancer risk.

**Methods:**

Databases, including MEDLINE, Embase, and Web of Science, were searched for all associated studies that evaluated associations between first-degree family history of female breast cancer and prostate cancer risk up to December 31, 2018. Information on study characteristics and outcomes were extracted based on the Preferred Reporting Items for Systematic Review and Meta-analysis (PRISMA) statement and Meta-analysis of Observational Studies in Epidemiology (MOOSE) guidelines. The quality of evidence was assessed using the GRADE approach.

**Results:**

Eighteen studies involving 17,004,892 individuals were included in the meta-analysis. Compared with no family history of female breast cancer, history of female breast cancer in first-degree relatives was associated with an increased risk of prostate cancer [relative risk (RR) 1.18, 95% confidence interval (CI) 1.12–1.25] with moderate-quality evidence. A history of breast cancer in mothers only (RR 1.19, 95% CI 1.10–1.28) and sisters only (RR 1.71, 95% CI 1.43–2.04) was associated with increased prostate cancer risk with moderate-quality evidence. However, a family history of breast cancer in daughters only was not associated with prostate cancer incidence (RR 1.74, 95% CI 0.74–4.12) with moderate-quality evidence. A family history of female breast cancer in first-degree relatives was associated with an 18% increased risk of lethal prostate cancer (95% CI 1.04–1.34) with low-quality evidence.

**Conclusions:**

This review demonstrates that men with a family history of female breast cancer in first-degree relatives had an increased risk of prostate cancer, including risk of lethal prostate cancer. These findings may guide screening, earlier detection, and treatment of men with a family history of female breast cancer in first-degree relatives.

## Background

Prostate cancer is the second most common cancer and the fifth leading cause of death in men worldwide [1, 2]. Cancer epidemiological data showed approximately 1,276,106 new prostate cancer cases and almost 358,989 cancer deaths worldwide in 2018 [2]. The cause of prostate cancer is complex and has not been fully determined. The possible risk factors are age, race, geography, family history, and genetic factors [3–5]. Among these risk factors, family history is a recognized risk factor for the development of prostate cancer [6, 7]. Patients with a family history of prostate cancer in first-degree relatives were 2.48 times more likely to develop prostate cancer than those without first-degree relatives with prostate cancer [8].

Approximately 35% of familial prostate cancer risk is explained by known genes [9, 10]. BRCA1 and BRCA2 are two major predisposition genes that induce hereditary breast and ovarian cancer [11, 12]. There is definite evidence that prostate cancer risk is increased in BRCA1 and BRCA2 mutation carriers ascertained by a family history of breast cancer [13]. BRCA1 mutation carriers increase the risk of prostate cancer in men aged < 65 years by 3.8-fold, and germline mutations in the BRCA2 gene increase prostate cancer risk by 8.6-fold [14, 15]. The mutation status of BRCA1/BRCA2 is closely related to the degree of prostate invasion, earlier death, and shorter survival time [15–17]. Moreover, previous observational studies have also reported that family history of breast cancer in first-degree relatives is associated with prostate cancer, including lethal prostate cancer [18, 19].

Recently, controversy came from several large-scale, high-quality analyses that attempted to analyse whether there was a correlation between the first-degree family history of female breast cancer and risk of prostate cancer. To better understand this issue, we performed a systematic review with meta-analysis of published literature that investigated the association between first-degree family history of female breast cancer and risk of prostate cancer.

## Methods

### Literature search and selection criteria

A systematic search in MEDLINE, Embase, and Web of Science was performed from the earliest publication date available until December 31, 2018. Additional studies were searched by checking the reference lists of relevant studies. The following search terms were used: ‘(prostate cancer OR prostate carcinoma OR prostate neoplasm) AND (breast cancer OR breast carcinoma OR breast neoplasm) AND (family history)’.

Studies were considered eligible if they (1) were published in the English language; (2) had full text available; (3) evaluated the relationship between first-degree family history of female breast cancer and prostate cancer risk; (4) provided risk estimates with confidence intervals (CIs) or available data to calculate these associations; and (5) were cohort, cross-sectional, and case-control studies.

### Data extraction and quality assessment

Two investigators independently extracted data using a standard data collection form. The data extracted from each study included the following: first author, publication year, study design, country of the study population, sample size, reported primary outcome, follow-up duration, hazard ratio or odds ratio, and relative risk and 95% confidence intervals (CIs) with and without adjustment and adjustment factors.

Two independent reviewers evaluated the quality of the included studies according to the Newcastle-Ottawa scale (NOS) [20]. The scale uses a ‘star’ rating system (maximum nine stars) to assess the quality of case-control and cohort studies including three aspects: selection of participants, comparability of study groups, and ascertainment of outcomes of interest [20]. If the study scored nine stars, it was considered to be of high quality. Studies with a score of seven or eight stars were considered to be of medium quality. However, if a study scored less than seven stars, it was considered to be of low quality. Any discrepancies in opinions were resolved by discussion with a third author.

### Grading the quality of evidence

The quality of evidence for outcomes was evaluated by two investigators independently using GRADEpro Guideline Development Tool (McMaster University, 2015, developed by Evidence Prime Inc., Hamilton, Canada; https://gradepro.org/). The quality of evidence was evaluated according to risk of bias, inconsistency, indirectness, imprecision of the results, and publication bias. The quality of evidence for the main outcome was classified into four grades: very low, low, moderate, and high.

### Statistical analysis

The primary outcome was relative risks for prostate cancer incidence. Subgroup analyses of the primary outcome were conducted based on the study design, region, and quality (adjustment vs no adjustment). For each study, risk ratio for prostate cancer with the 95% CI was computed. The random effects model was used to compute the pooled risk ratio. Heterogeneity between studies was evaluated using the chi-square-based Q test and I^2^ metric. If *P* < 0.10 and I^2^ > 50%, the heterogeneity was considered statistically significant. The significance of the summary RR was assessed using the Z-test, and a *P*-value < 0.05 was considered as statistically significant. A sensitivity analysis was conducted to evaluate the stability of the results by excluding individual studies each time. Funnel plots and Begg’s and Egger’s tests were used to investigate the potential publication bias. All statistical analyses were conducted using Stata software version 12.0 (Stata Corporation, College Station, Texas, USA).

## Results

### Retrieved studies and characteristics

**Fig. 1:**
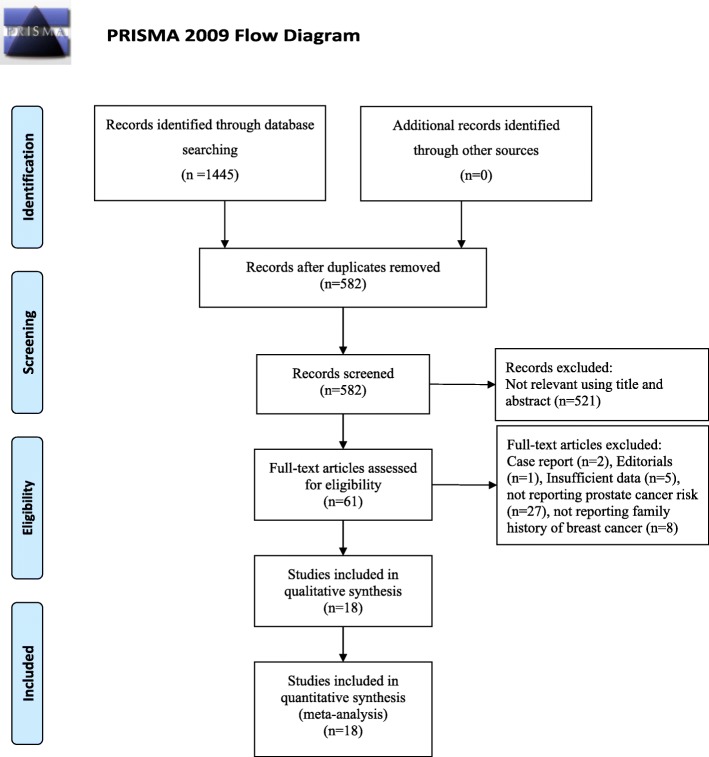
Flow chart of study selection

The systematic search of articles published before December 31, 2018, identified 1554 articles. After screening titles and abstracts, we obtained 61 study reports for full-text review. After a full-text review, we finally included 18 published reports comprising 17,004,892 individuals for analysis [19, 21–37] (Fig. 1). Overall, six were cohort studies, 11 were case-control studies, and one was a cross-sectional study. Ten of these studies were based in America, 5 in Europe, and 3 in Asia. A history of breast cancer in first-degree relatives was reported in 13 studies, in mothers only in 11 studies, and in sisters only in 10 studies. The articles were published between 1992 and 2018. The detailed characteristics of all included studies are shown in Table 1. The quality of studies based on the NOS score is presented in Table 2. Most studies were of medium to high quality (score ≥ 7). Six case-control studies were of low quality.

**Table 1:** Characteristics of studies included in the meta-analysis

Author	Year	Country	Study disgn	Follow up duration	Sample size	Exposure	Measure of effect	RR (prostae cancer risk) (95% CI)	Adjustment factors
Tulinius	1992	Iceland	Cohort	1955–1988	29,725	Mother with BCa	RR	1.40(0.51,3.05)	–
						Sister with BCa		1.29(0.9,1.79)	
						Daughter with BCa		1.45(1.02,2.00)	
Goldgar	1994	USA	Cohort	1952–1992	656,017	First degree relatives with BCa	RR	1.23(1.1,1.3)	–
Hayes	1995	USA	Case-control	–	Case: 981 Control: 1315	First degree relatives with BCa	OR	1.3(0.9,1.9)	Socio-economic status, based upon usual occupation,education, income, and marital status
						Mother with BCa		1.0(0.6,1.7)	
						Sister with BCa		1.8(1.1,3.0)	
Isaacs	1995	USA	Case-control	–	Case: 690 Control: 683	Mother with BCa	OR	2.05(1.01,4.14)	Age
						Sister with BCa	OR	1.53(0.78,3.00)	
McCAHY	1996	UK	Case-control	–	Case:209 Control:322	First degree relatives with BCa	OR	1.69(0.9,3.15)	–
Glover	1998	Jamaica	Case-control	–	Case: 263 Control: 263	First degree relatives with BCa	OR	0.89(0.46,1.71)	–
Rodriguez	1998	USA	Cohort	1982–1994	480,802	First degree relatives with BCa	RR	1.16(1.01,1.33)	Age, race, years of education, number of sisters and number of sisters older than 50 years of age, Jewish religion, BMI, physical activity, vegetable and fat intake, smoking status, and previous vasectomy
						Mother with BCa		1.34(1.11,1.62)	
						Sister with BCa		0.97(0.78,1.20)	
						History of BCa diagnosis at age<50		1.23(0.94,1.62)	
						History of BCa diagnosis at age>50		1.16(0.98,1.37)	
Kalish	2000	USA	Cohort	1987–1997	1156	Mother with BCa	RR	1.18(0.51,2.43)	–
Bai	2005	China	Case-control	–	Case:238 Control:471	First degree relatives with BCa	OR	2.04 (0.75, 5.51)	Age, vasectomy history
						Mother with BCa		2.01 (0.28, 14.38)	
						Sister with BCa		4.03 (0.73, 22.14)	
						Daughter with BCa		1.01 (0.18, 5.54)	
Negri	2005	Italy	Case-control	–	Case:1294 Control:2820	First degree relatives with BCa	OR	1.20(0.8,1.8)	Age, study centre, period of interview, education, occupational physical activity at 30–39 years of age and no of siblings (or sisters or brothers when appropriate)
Beebe-Dimmer	2006	USA	Case-control	–	Case:121 Control:179	Mother with BCa	OR	0.52 (0.10,2.69)	Age
						Sister with BCa		3.80 (1.57–9.22)	
						Daughter with BCa		1.01 (0.19–5.28)	
Suzuki	2007	Japan	Case-control	–	Case: 257 Control: 28,125	First degree relatives with BCa	OR	3.6 (1.1–11.7)	Smoking history, drinking, BMI, exercise habit, and referral pattern to the hospital
Chen	2008	USA	Cohort	1986–2004	51,529	First degree relatives with BCa	RR	1.30(1.13,1.49)	Ethnicity, BMI, total calories, vigorous activity, cigarette smoking, and consumption of tomato sauce, calcium, alpha linolenic fatty acid, fish, and red meat
						Mother with BCa		1.24(1.06,1.45)	
						Sister with BCa		1.19(0.98,1.45)	
Mori	2011	Japan	Case-control	–	Case:142 Control:468	Mother or sister with BCa	OR	2.70(1.12,6.49)	–
Thomas II	2012	USA	Cross section	–	8122	Frist degree relatives with BCa	OR	1.04(0.84,1.29)	Age, race, PSA, BMI, TRUS volume, geographic region, DRE findings and treatment arm
						Mother with BCa		1.07(0.8,1.42)	
						Sister with BCa		1.30(0.95,1.78)	
Frank	2017	Sweden	Cohort	1958–2012	15,700,000	Frist degree relatives with BCa	RR	1.12(1.08,1.16)	Sex, age group, calendar period, residential area, and socioeconomic status
Barber	2018	USA	Cohort	1996–2012	37,002	Frist degree relatives with BCa	HR	1.21(1.1,1.34)	Age, race, BMI, smoking status, PSA screening, PSA testing intensity, alcohol intake, vigorous physical activity, total energy intake, consumption of tomato sauce, and red meat
						Mother with BCa		1.14(1.01,1.27)	
						Sister with BCa		1.20(1.04,1.39)	
Lamy	2018	France	Case-control	–	Case:819 Control:879	First degree relatives with BCa	OR	1.13(0.84,1.52)	Age, ethnic origin, number of first-degree female relatives and famili history of prostate cancer in first-degree relatives
						Mother with BCa		1.04(0.71,1.52)	
						Sister with BCa		1.10(0.72,1.68)	
						Daughter with BCa		15.26(1.95,120)	
						History of BCa diagnosis at age<50		1.79(1.09,2.94)	
						History of BCa diagnosis at age>50		0.88(0.61,1.27)	

BCa: breast cancer; PCa: prostate cancer; RR: Relative risk; OR: odds ratio; HR: hazard ratio

**Table 2:** Quality assessment of included studies

Author	Year	Selection	Comparability	Exposure	Total
Tulinius	1992	★★★	★★	★★	7
Goldgar	1994	★★★	★★	★★	7
Hayes	1995	★★★	★★	★★	7
Isaacs	1995	★★	★★	★★	6
McCAHY	1996	★★	★	★★★	6
Glover	1998	★★	★★	★★	6
Rodriguez	1998	★★★	★★	★★★	8
Kalish	2000	★★★	★★	★★	7
Bai	2005	★★	★★	★★	6
Negri	2005	★★★	★★	★★	7
Beebe-Dimmer	2006	★★	★★	★★	6
Suzuki	2007	★★	★★	★★	6
Chen	2008	★★	★★	★★★	7
Mori	2011	★★★	★★	★★	7
Frank	2017	★★★	★★	★★★	8
Barber	2018	★★★	★★	★★★	8
Lamy	2018	★★★	★★	★★★	8

### Associations between family history of breast cancer and risk of prostate cancer

**Fig. 2:**
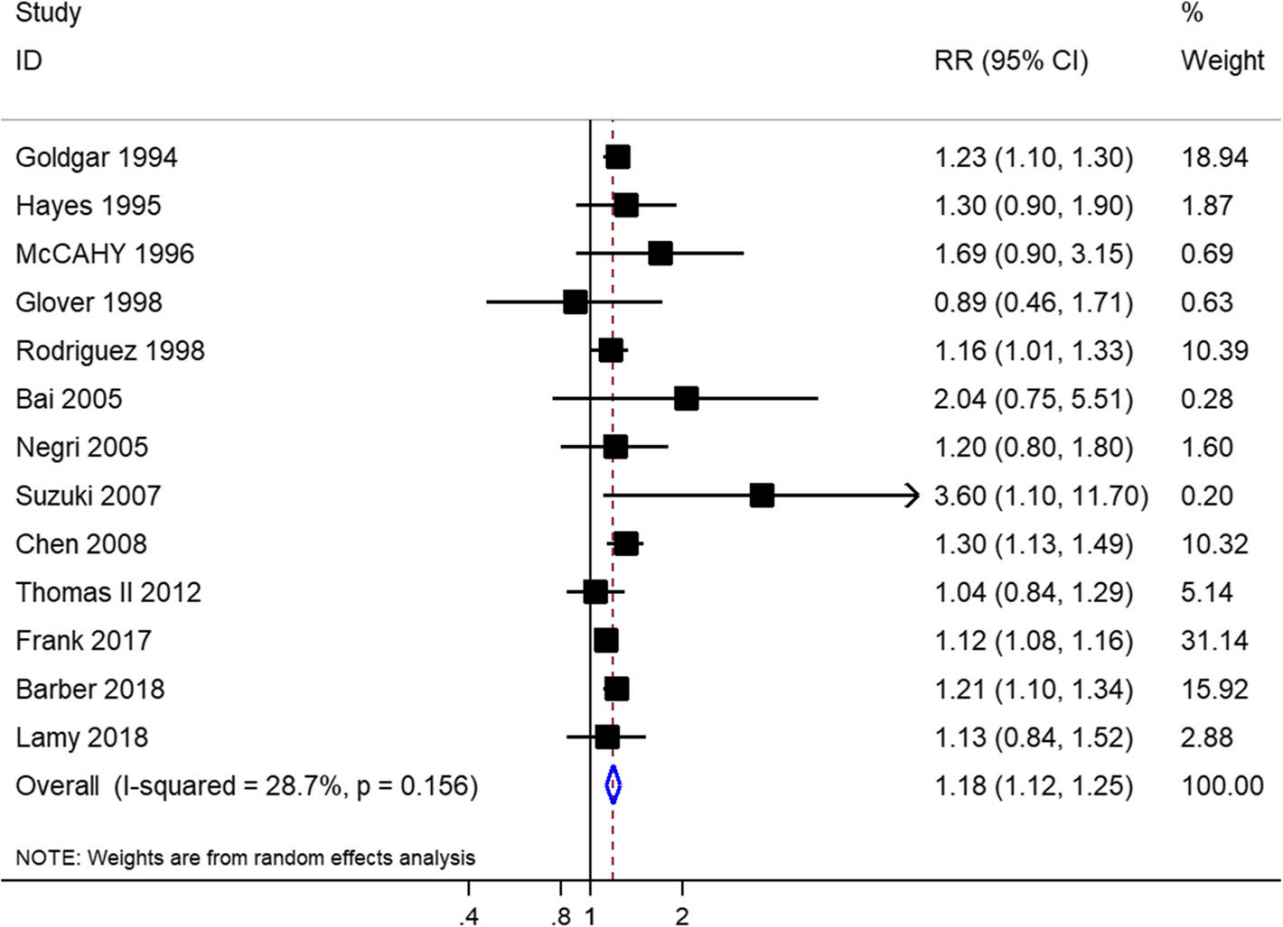
Forest plot of studies reporting association between family history of female breast cancer in first-degree relatives and prostate cancer risk

Eighteen studies with 17,004,892 individuals in total evaluated the association between family history of breast cancer and risk of prostate cancer. Of these, 13 studies with a total of 16,971,728 individuals evaluated the association between family history of female breast cancer in first-degree relatives and risk of prostate cancer. The history of female breast cancer in first-degree relatives was significantly associated with prostate cancer risk (RR = 1.18, 95% CI = 1.12–1.25, I^2^ = 28.70%) (Fig. 2), with moderate-quality evidence (Table 3). This increased risk with family history of female breast cancer persisted in studies that adjusted for potential confounders (adjusted RR, 1.17; 95% CI, 1.10–1.24; I^2^ = 25.30%) (Table 4). When we stratified our analysis by study design, a significantly increased association was observed in the pooled cohort studies (RR, 1.17; 95% CI, 1.10–1.25; I^2^ = 48.90%) and pooled case-control studies (RR, 1.23; 95% CI, 1.14–1.33; I^2^ = 0.00%) (Table 4). Subgroup analyses based on the study region showed that a family history of female breast cancer was significantly associated with prostate cancer risk in America, Europe, and Asia (Table 4). Moreover, this increased prostate cancer risk was not observed in first-degree relatives with a breast cancer diagnosis at age < 50 years (RR = 1.40, 95% CI = 0.99–1.98, I^2^ = 40.00%) and ≥ 50 (RR = 1.06, 95% CI = 0.83–1.37, I^2^ = 45.00%) (Table 4).

**Table 3:** GRADE assessment of quality of the body of evidence, and summary of findings

Association studied	No. of studies	Design	Risk of bias	Inconsistency	Indirectness	Imprecision	Factors that can increase quality of evidence	Pooled effect estimate	Quality
Family history of BCa in first degree relatives and risk of PCa	13	Observational study	Not serious	Not serious	Not serious	Not serious	All plausible confounding would reduce a demonstrated effect	1.14(1.10,1.18)	⨁⨁⨁◯MODERATE
Family history of BCa in mothers and risk of PCa	11	Observational study	Not serious	Not serious	Not serious	Not serious	All plausible confounding would reduce a demonstrated effect	1.19(1.10,1.28)	⨁⨁⨁◯MODERATE
Family history of BCa in sisters and risk of PCa	10	Observational study	Not serious	Not serious	Not serious	Not serious	All plausible confounding would reduce a demonstrated effect	1.16(1.06,1.27)	⨁⨁⨁◯MODERATE
Family history of BCa in daughters and risk of PCa	4	Observational study	Not serious	Not serious	Not serious	Not serious	All plausible confounding would reduce a demonstrated effect	1.74(0.74,1.42)	⨁⨁⨁◯MODERATE
Family history of BCa in first degree relatives and risk of lethal PCa	2	Observational study	Not serious	Not serious	Not serious	Not serious	None	1.18(1.04,1.34)	⨁⨁◯ ◯LOW
Family history of BCa in mothers and risk of lethal PCa	2	Observational study	Not serious	Not serious	Not serious	Not serious	None	1.35(1.14,1.61)	⨁⨁◯ ◯LOW
Family history of BCa in sisters and risk of lethal PCa	2	Observational study	Not serious	Not serious	Not serious	Not serious	None	1.02(0.84,1.23)	⨁⨁◯ ◯LOW

BCa: breast cancer; PCa: prostate cancer

**Table 4:** Subgroup analysis for studies included in the analysis

Prostate cancer risk	No. of studies	Pooled RR (95% CI)	I^2^ statistics (%)	*P*-value for the heterogeneity Q test
First degree relatives with BCa	13	1.18(1.12,1.25)	28.70%	0.156
Cohort	5	1.19(1.12,1.26)	53.70%	0.071
Case-control	7	1.26(1.04,1.53)	6.90%	0.375
Cross section	1	1.04(0.84,1.29)	–	–
European	4	1.12(1.08,1.16)	0.00%	0.624
American	7	1.21(1.15,1.27)	0.00%	0.618
Asian	2	2.58(1.21,5.54)	0.00%	0.472
Adjustment for other factors				
Yes	10	1.17(1.10,1.24)	25.30%	0.210
No	3	1.23(1.13,1.34)	0.00%	0.383
BCa diagnosis at age ≥ 50	2	1.06(0.83,1.37)	45.00%	0.179
BCa diagnosis at age <50	2	1.40(0.99,1.98)	40.00%	0.195
Mother with BCa	11	1.19(1.10,1.28)	0.00%	0.686
Cohort	5	1.21(1.11,1.31)	0.00%	0.671
Case-control	5	1.14(0.85,1.54)	7.30%	0.365
Cross section	1	1.07(0.80,1.43)	–	–
European	2	1.09(0.77,1.54)	0.00%	0.549
American	8	1.19(1.10,1.29)	0.00%	0.480
Asian	1	2.01(0.28,14.40)	–	–
Adjustment for other factors				
Yes	8	1.19(1.10,1.28)	0.10%	0.428
No	3	1.32(0.75,2.32)	0.00%	0.873
Sister with BCa	10	1.25(1.09,1.44)	43.00%	0.071
Cohort	4	1.15(1.04,1.28)	8.40%	0.351
Case-control	5	1.75(1.14,2.70)	50.00%	0.091
Cross section	1	1.30(0.95,1.78)	–	–
European	2	1.21(0.93,1.58)	0.00%	0.567
American	7	1.26(1.07,1.50)	55.60%	0.035
Asian	1	4.03(0.73,22.19)	–	–
Adjustment for other factors				
Yes	8	1.24(1.06,1.44)	48.80%	0.057
No	2	1.66(0.66,4.18)	39.20%	0.200
Daughter with BCa	4	1.74(0.74,4.12)	43.70%	0.149
Cohort	1	1.45(1.04,2.03)	8.40%	0.351
Case-control	3	2.27(0.44,11.75)	62.50%	0.046
European	2	3.74(0.39,35.97)	79.50%	0.027
American	1	1.01(0.19,5.28)	–	–
Asian	1	1.01(0.18,5.54)	–	–
Adjustment for other factors				
Yes	2	3.66(0.26,52.14)	75.30%	0.044
No	2	1.43(1.03,1.99)	0.00%	0.685

BCa: breast cancer; PCa: prostate cancer

**Fig. 3:**
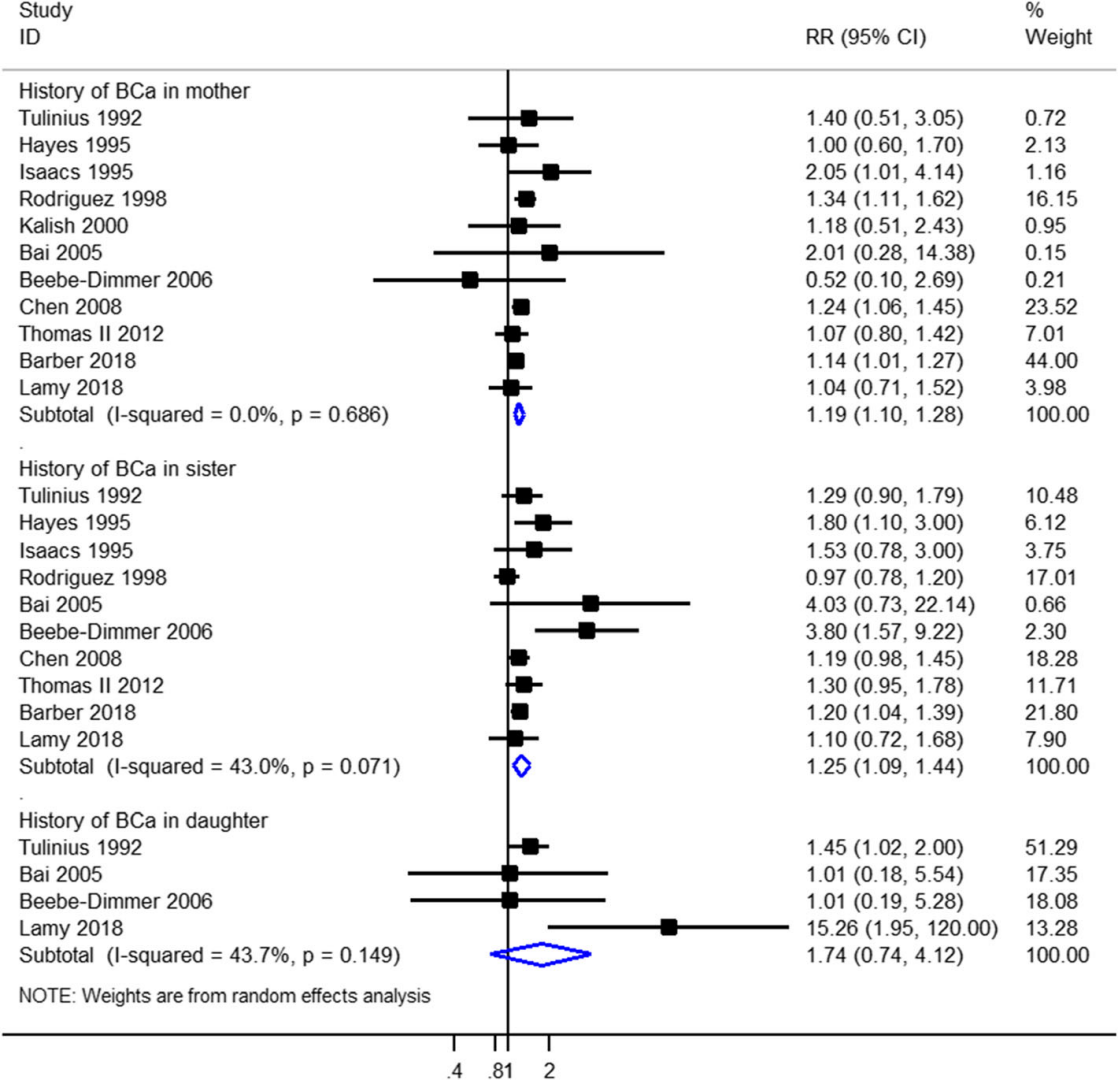
Forest plot of studies reporting association between family history of female breast cancer and prostate cancer risk by source of family history

A history of breast cancer in mothers only was reported in 11 studies (614,712 participants). A family history of breast cancer in mothers only was associated with prostate cancer incidence (RR = 1.19, 95% CI = 1.10–1.28, I^2^ = 0.00%) with moderate-quality evidence (Fig. 3, Table 3). This increased risk with family history of breast cancer persisted in studies that adjusted for potential confounders (adjusted RR, 1.19; 95% CI, 1.10–1.28; I^2^ = 0.10%) (Table 4). When we stratified our analysis by study design, there was a statistically significant increased association in the five pooled cohort studies (RR, 1.21; 95% CI, 1.11–1.31; I^2^ = 0.00%), but no association between history of breast cancer in mothers only and prostate cancer risk was observed in the five pooled case-control studies (RR = 1.14, 95% CI = 0.85–1.54, I^2^ = 7.30%) (Table 4). Subgroup analyses based on the study region showed that a statistically significant increased association between history of breast cancer in mothers only and prostate cancer risk was observed in America, but not in Europe and Asia (Table 4).

A history of breast cancer in sisters only was reported in 10 studies (613,556 participants). A family history of breast cancer in sisters was associated with prostate cancer (RR =1.25, 95% CI = 1.09–1.44, I^2^ = 43.00%) with moderate-quality evidence (Fig. 3, Table 3). This increased risk with family history of breast cancer persisted in studies that adjusted for potential confounders (adjusted RR, 1.24; 95% CI, 1.06–1.44; I^2^ = 48.80%) (Table 4). Subgroup analyses based on the study design showed that a consistent result was observed in the pooled cohort studies (RR, 1.15; 95% CI, 1.04–1.28; I^2^ = 8.40%) and pooled case-control studies (RR, 1.75; 95% CI, 1.14–2.70; I^2^ = 50.00%) (Table 4). When we stratified our analysis by the study region, there was a statistically significant association in America, but no association between history of breast cancer in sisters only and prostate cancer risk in Europe and Asia (Table 4).

A history of breast cancer in daughters only was reported in 4 studies (32,432 participants). A family history of breast cancer in daughters only was not associated with prostate cancer (RR = 1.74, 95% CI = 0.74–4.12, I^2^ = 43.70%) with moderate-quality evidence (Fig. 3, Table 3). Similarly, no increased risk with family history of breast cancer in daughters only was observed in studies that adjusted for potential confounders (RR, 3.66; 95% CI, 0.26–52.14; I^2^ = 75.30%) (Table 4). Subgroup analyses based on the study design showed that a statistically significant increased association between history of breast cancer in daughters only and prostate cancer risk was observed in cohort studies, but not in case-control studies (Table 4). When we stratified our analysis by study region, no significant association was observed in America, Europe, and Asia (Table 4).

### Associations between family history of female breast cancer and risk of lethal prostate cancer

**Fig. 4:**
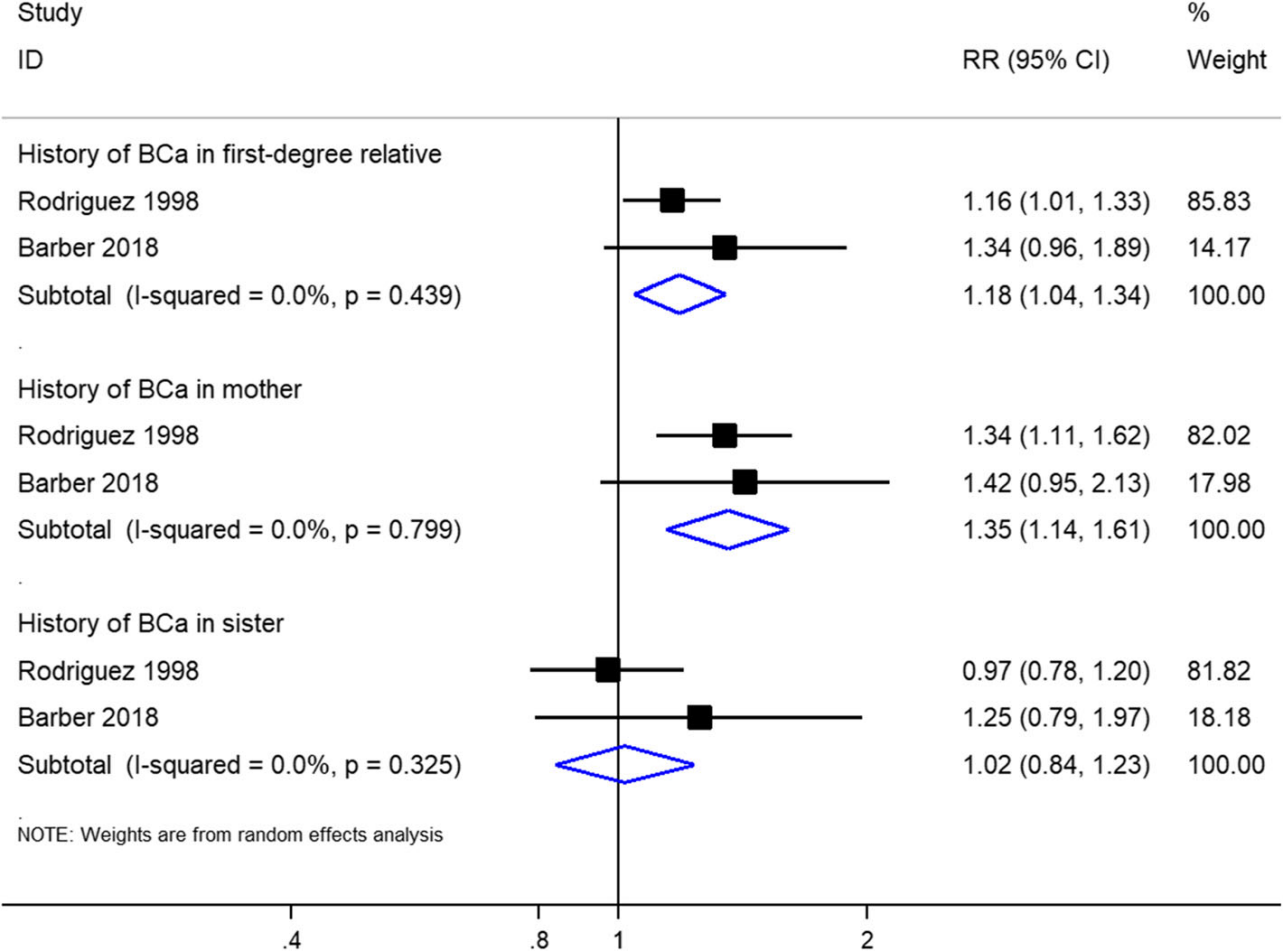
Forest plot of studies reporting association between family history of female breast cancer and lethal prostate cancer risk

Two studies, including a total of 517,804 individuals, evaluated the association between family history of female breast cancer and risk of lethal prostate cancer. There was no significant heterogeneity among the studies (I^2^ = 0.00%). The increased risk of lethal prostate cancer was observed in individuals with family history of female breast cancer in first-degree relatives and in mothers only; however, no association was found between family history of breast cancer in sisters only and risk of lethal prostate cancer, with low-quality evidence (Fig. 4).

### Sensitivity analysis and publication bias

**Fig. 5:**
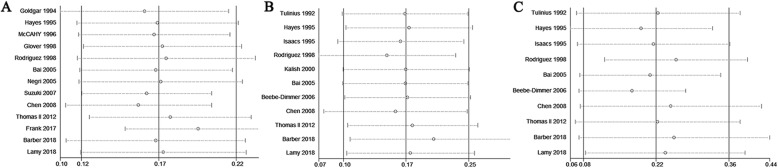
Sensitivity analysis diagrams for each study used to assess the association between family history of female breast cancer and prostate cancer risk. (**a**. Family history of breast cancer in first-degree relatives; **b**. Family history of breast cancer in mother only; **c**. Family history of breast cancer in sister only)

**Fig. 6:**
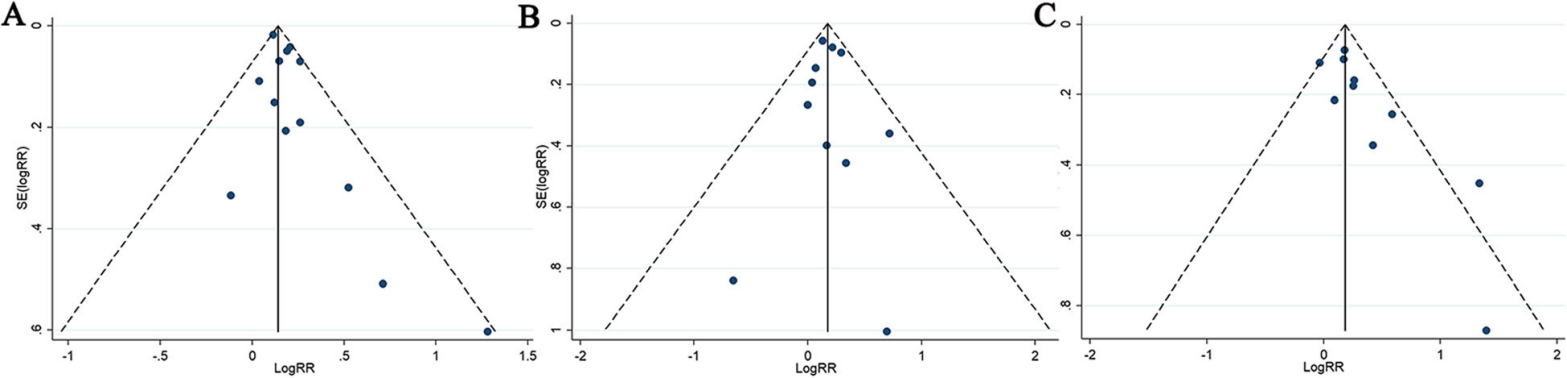
Funnel plots of the studies assessing the association between family history of female breast cancer and prostate cancer risk. (**a**. Family history of breast cancer in first-degree relatives; **b**. Family history of breast cancer in mother only; **c**. Family history of breast cancer in sister only)

A sensitivity analysis was conducted for prostate cancer risk by excluding individual studies each time, and the results showed no individual study influenced the overall RRs (Fig. 5), indicating the results of this meta-analysis are relatively stable. Some publication bias for the history of breast cancer in sisters only was observed in the results based on Egger’s tests (*P* = 0.037) and funnel plots (Table 5, Fig. 6). No publication bias was observed based on visual inspection of funnel plots or Begg’s and Egger’s test for history of female breast cancer in first-degree relatives and mothers only (Table 5, Fig. 6).

**Table 5:** Publication bias test for the history of female breast cancer and risk of prostate cancer

Exposure		Egger test		Begg
	Coefficient	P	95% CI	
First degree relatives with female BCa	0.837	0.052	−0.008 to 1.683	0.360
History of BCa in mother only	0.072	0.863	−0.887 to 1.030	0.640
History of BCa in sister only	1.669	0.024	0.283 to 3.056	0.049

BCa: breast cancer; PCa: prostate cancer

## Discussion

Eighteen studies involving 17,004,892 participants met the inclusion criteria and were eventually included in our meta-analysis. The findings of this review suggest that prostate cancer risk was increased in individuals with a family history of female breast cancer in first-degree relatives, in mothers only and sisters only. Importantly, we observed increased lethal prostate cancer risks in individuals with family history of female breast cancer in first-degree relatives and mothers only, but not in sisters only. These findings are of great significance because the underlying pathogenesis of prostate cancer is still unknown and may help in screening, earlier diagnosis, and management of prostate cancer.

Prostate cancer pathogenesis includes both heritable and environmental causation [38–40]. Family history was one of the most important factors in prostate cancer [41, 42]. Previous meta-analyses observed more than twofold increased prostate cancer risk in men who have a first-degree relative with prostate cancer [8, 43]. A family history of breast cancer has also been considered as a possible risk factor for prostate cancer [19, 26]. A family history of breast cancer has previously been associated with prostate cancer risk in a cohort study based on the Swedish Family-Cancer Database [21]. Similarly, a cohort study conducted by Barber et al. showed that men with first-degree relatives diagnosed with breast cancer are 21% more likely to develop prostate cancer than normal individuals and men with a family history of prostate and breast cancers are also at higher risk [19]. However, several studies found no association between family history of breast cancer and risk of prostate cancer. Thomas II et al. observed that a family history of breast cancer alone was not related to increased prostate cancer risk [24]. Bai et al. reported that risk of prostate cancer was not significantly related to family history of breast cancer in China [34]. Moreover, several studies have estimated the effect of family history of breast cancer in mothers only, sisters only, and daughters only with varying results. A prospective study on 37,002 US men in the Health Professionals Follow-up Study showed that a family history of breast cancer in mothers only and sisters only was significantly associated with increased prostate cancer risk [19], and the results were consistent with those of two cohort studies [18, 26]. We also observed a positive association between history of breast cancer in daughters only and increased prostate cancer risk in cohort and case-control studies [23, 28]. However, other studies reported no significant association between prostate cancer risk and family history of breast cancer in mothers only, sisters only, and daughters only [24, 27]. This difference between studies may be due to the study design, sample size, nationalities, or study regions. Thus, more high-quality studies are needed to assess the associations.

In the subgroup meta-analyses based on the study region, a family history of female breast cancer in first-degree relatives was associated with prostate cancer risk in Europe, America, and Asia. A family history of breast cancer in mothers only and sisters only was associated with prostate cancer risk in America, while no significant association was found in Europe and Asia. A family history of breast cancer in daughters only was not associated with prostate cancer risk in Europe, America, and Asia. However, these results need to be interpreted with caution because the number of studies reported in Europe and Asia was relatively small; thus, more studies are warranted to further investigate the potential relationships between family history of female breast cancer and prostate cancer risk in Europe and Asia. In the subgroup meta-analyses based on the study design, a family history of breast cancer in first-degree relatives and sisters only was associated with prostate cancer risk in cohort and case-control studies. A family history of breast cancer in mothers only and daughters only was associated with prostate cancer risk in cohort studies, but not in case-control studies. It is considered that these negative associations were attributed to the limited number of studies included in the meta-analysis.

In our analysis, we observed that men with a family history of female breast cancer have a higher risk of prostate cancer, including lethal prostate cancer. The underlying mechanisms of the associations are still unclear. A common gene alteration may be responsible for the clustering of prostate and breast cancer. BRCA1 and BRCA2 gene mutations, confirmed to be linked to breast cancer in families [44, 45], confer a 3.8- and 8.6-fold increased risk of developing prostate cancer, respectively [14, 15]. BRCA2 carriers are associated with poor prognosis and more aggressive form in prostate cancer [46, 47]. In addition to BRCA1 and BRCA2 genes, previous studies supported the contribution of other undetermined genetic factors to the aetiology and prognosis of prostate cancer in breast cancer-prone families [48–50]. Further studies are needed to explore the mechanism of the relationship between family history of female breast cancer and lethal prostate cancer risk and provide further data on the incidence and prognosis of prostate cancer in individuals with a family history of female breast cancer.

As the number of studies increased, we could perform multiple subgroup analyses to assess heterogeneity and publication bias. To our knowledge, our study was the first systematic literature review with a meta-analysis to evaluate the relationship between family history of female breast cancer and prostate cancer risk. The large sample size is another important strength of this study. The heterogeneity and publication bias in this meta-analysis are small. Moreover, we rigorously used the GRADE approach to assess quality of evidence for the main outcome. However, this study has several limitations. First, there were too few studies to draw a definitive conclusion for the risk of lethal prostate cancer in men with a family history of breast cancer. More prospective cohort studies that evaluate the incidence and prognosis of prostate cancer in men with a family history of female breast cancer are needed. Second, the results showed that the risk of prostate cancer was not significant in individuals with family history of female breast cancer in first-degree relatives diagnosed with breast cancer at the age of < 50 and ≥ 50 years. The results need to be interpreted with caution because only two studies reported these associations in these analyses. Finally, due to the lack of relevant information in the included studies, we did not estimate the risk of early-onset prostate cancer in men with a family history of female breast cancer.

## Conclusions

Therefore, the results of this meta-analysis indicate that a family history of female breast cancer in first-degree relatives was associated with an increased risk of prostate cancer, including lethal prostate cancer. These findings reinforce the importance of family history of female breast cancer in prostate cancer risk, beyond the roles of family history of prostate cancer. Further detailed work is needed to better investigate the mechanism of these associations and assess the association between family history of female breast cancer and prostate cancer progression and prognosis.

## Data Availability

All data generated or analysed during this study are included in this manuscript.

## Abbreviations

BCa: Breast cancer
BRCA1: Breast Cancer Susceptibility Gene 1
BRCA2: Breast Cancer Susceptibility Gene 1
CI: Confidence interval
CIs: Confidence intervals
HR: Hazard ratio
MOOSE: Meta-analysis of Observational Studies in Epidemiology
NOS: Newcastle-Ottawa scale
OR: Odds ratio
PCa: Prostate cancer
PRISMA: Preferred Reporting Items for Systematic Review and Meta-analysis
RR: Relative risk
